# Soccer Scores, Short-Term Mood and Fertility

**DOI:** 10.1007/s10680-021-09576-2

**Published:** 2021-04-14

**Authors:** Fabrizio Bernardi, Marco Cozzani

**Affiliations:** European University Institute, Via dei Roccettini 9, 50014 Fiesole, Firenze Italy

**Keywords:** Fertility, Short-term mood, Soccer, Causal effect

## Abstract

Previous research has shown that seemingly irrelevant events such as unexpected outcomes in sporting events can affect mood and have relevant consequences for episodes of crime and violence, investing behavior and political preferences. In this article, we test whether mood shocks associated with unexpected results in soccer matches in Spain affect fertility. We use data on betting odds and actual scores to define mood shocks and link them to births by month and province in Spain, between 2001 and 2015. We find that unexpected losses of local teams lead to a small decrease in the number of births nine months thereafter. The effect is larger for more unexpected losses, in those provinces with the largest amount of support for the local team and robust to a number of placebo tests. We argue that these results are consistent with the gain–loss asymmetry predicted by prospect theory.

## Introduction

There is a growing body of research suggesting that seemingly irrelevant events might affect mood and subsequently individual decisions and behaviors (Alengoz et al. 2017; Bassi et al. 2013; Hirshleifer and Shumway 2003; Kamstra et al. 2003; Schwarz and Clore 1983). Within this literature, some studies have shown that intense emotions elicited by sport events affect risk-taking economic decisions (Edmans et al. 2007), political preferences and opinions (Busby et al. 2017; Healy et al. 2010), criminal behaviors (Munyo and Rossi 2013), domestic violence (Card and Dahl 2011) and, in the case of pregnant women, their newborns’ birth weight (Duncan et.al. 2017). There is also recurrent anecdotal evidence that sport events can influence fertility behaviors. For instance, newspapers reported an increase in the number of births in Iceland nine months after the country unexpectedly beat England in the 2016 UEFA Europe Cup; in Germany in 2007 after its initially successful performance in the FIFA 2006 World Cup; and in England in 2003 after it reached the quarter-finals of FIFA World Cup in 2002 (Connolly 2007; Gibson 2017; Womack 2003).

Two recent academic articles have investigated whether sport results affect fertility, but the findings are not conclusive. Montesinos et al. (2013) document a spike in fertility in the Barcelona area nine months after a dramatic goal scored by Iniesta against Chelsea in the last minute of the semifinal of the UEFA Champions League in 2009, in line with anecdotal evidence reported in the Spanish news.^1^ On the other hand, Hayward and Rybińska (2017) find that the Super Bowl does not produce an increase in the number of birth in the counties of winning teams nine months later, contrary to the anecdotal evidence in the news and a widely broadcasted NFL announcement.^2^

In this article, we test the hypothesis that mood shocks influence fertility behavior, by studying the effect of unexpected results of soccer games in Spain on the number of births nine months thereafter. In Spain, soccer is by far the most popular sport, with a large number of both male and female followers who strongly identify with their local team. In our empirical analyses, we link the universe of monthly births in Spain between 2001 and 2015, to the betting odds of games played in the Spanish first division soccer league nine months before in a given province.

We make two contributions to the literature. First, at the theoretical level, we apply prospect theory to explain the effect of short-term mood variations on fertility (Kőszegi and Rabin 2006). More precisely, we refer to a model of reference-dependent utility. We argue that mood depends on deviations around a rationally expected reference point and that there might be an asymmetry in the effect of unexpected gains and losses with respect to the reference point, with the losses entailing larger negative shocks on mood and, thus, on fertility.

Second, we use an innovative research design based on big data that enable us to provide a causal estimate of the effect of mood fluctuations on fertility. There is now some evidence that happier people are more likely to have children and conversely that stress and poor mood negatively affect childbearing (Cetre et al. 2016; Greil 1997; Le Moglie et al. 2015; Margolis and Myrskylä 2011; Parr 2010). A major challenge in this stream of research is to show that subjective well-being and mood causally affect childbearing. This is because mood fluctuations are typically unobservable and mostly driven by events that are also related to other determinants of fertility. To overcome the endogeneity of mood fluctuations, we study mood shocks arising from soccer results. We use data on betting odds of soccer games and compare them to actual scores in order to measure exogenous shocks around expected outcomes. Conditional on betting markets aggregating information efficiently, deviations from expected outcomes, as predicted by the betting odds, and the associated mood changes are random (Card and Dahl 2011; Munyo and Rossi 2013). Our estimates of the effect of mood on fertility have, therefore, a causal interpretation.

In our empirical analysis, we control for province and month via year fixed effects and find that unexpected losses of a team of a given province lead to approximately a 0.8 percent decrease in the number of births in that same province nine months later. This effect is small in size but robust to a number of additional specifications and placebo tests. In the conclusions, we discuss the theoretical and substantive relevance of this finding.

## Sport, Mood and Fertility: Possible Explanations

We discuss two different explanations of how sport results might affect mood and fertility. Although the logic of these explanations is similar as both argue that sport events affect mood and consequently fertility behavior, they critically differ in their prediction of the symmetry/asymmetry in the effect of victory and defeats on mood and then fertility.

### Celebratory Intercourse Versus Sorrowful Abstention?

A common explanation for the link between sport results, mood and fertility lies in what Hayward and Rybińska (2017) label “celebratory intercourse.” For instance, in order to explain the so-called Iniesta generation (i.e., the spike in number of births in the Barcelona area nine months after Iniesta’s goal against Chelsea in 2009) Montesinos et al. (2013, p. 6) argue that “heightened euphoria following a victory can cultivate hedonic sensations that result in intimate celebrations, of which unplanned births may be a consequence.” Discussing the Super Bowl Babies, Hayward and Rybińska (2017) do not limit their explanation to a rise in unplanned births. They argue for a more general heightened propensity for intercourse, also among the couples who were already planning to have a baby. The plausible underlying physiological mechanism given is that the positive feelings after one’s team wins go hand in hand with some hormonal change that, in turn, increase sexual desire (Casto and Edwards 2016; van der Meij et al. 2012a, b). There is indeed evidence that the vicarious experience of winning or losing in the context of political election or sport events is associated with changes in testosterone and cortisol, generating a winner–loser effect of hormonal fluctuations (Bernhardt et al. 1998; Stanton et al. 2009; van der Meij et al. 2012a, b). For instance, a study found that after the 2004, 2006 and 2008 US elections, those states that won the election showed an increase in pornography-seeking behaviors, whereas losing states showed a significant decrease (Markey and Markey 2010). In sports, Bernhardt et al. (1998) have investigated testosterone levels of male Brazilian and Italian fans after the 1994 World Cup soccer final and found that testosterone level rose in fans who won, whereas it decreased in fans that experienced a loss.

Previous studies on the effects of sport outcomes on fertility have not, however, elaborated on the consequences of defeats and thus of negative emotions. For every winning team and euphoric group of fans, there is a corresponding defeated team and group of sorrowful supporters. In this respect, “sorrowful abstention” driven by negative mood is the opposite explanation to “celebratory intercourse” proposed (Hayward and Rybińska 2017). One can then hypothesize that negative mood shock induced by a defeat has a negative effect on fertility because it reduces the probability of intercourse.

### Prospect Theory

Previous studies on the effect of unexpected emotional cues on individual behavior have been based a model of reference-dependent utility (Card and Dahl 2011; Munyo and Rossi 2013). According to this model, individuals’ emotions (euphoric or sorrowful mood) vary depending on gains or losses around some reference point for the outcome of interest (Kőszegi and Rabin 2006). It is not the simple level of gain or loss that drives individual mood, but the level compared to prior expectations of the outcome. Based on past experiences and the information at hand, individuals expect that the outcome of interest will be of *x*. The level *x* then becomes the reference point that is used to evaluate the actual outcome. Results above *x* are associated with an increase in utility and, thus, good mood, while results below *x* are associated with a loss in utility and, accordingly, to a bad mood. The crucial additional element of prospect theory is loss aversion: the idea that losses resonate more than same-sized gains for individual utility (Kahneman and Tversky 1979). This means that a gain *k* with respect to the reference point *x* fosters less positive emotions, when compared to the negative emotions associated with the same size loss *k*. The notion of loss aversion is motivated by experimental evidence that consistently shows that subjects take decision weighting more losses than gain (Barberis 2013) but also by observational findings suggesting, for instance, that well-being is more sensitive to income losses than to equivalent income gains (Boyce et al. 2013). Recent research in psychology and neuroscience suggests that that loss aversion is linked to neurohormonal mechanisms, and in particular to the functioning of the amygdala and noradrenaline (Sokol-Hessner and Rutledge 2019).

Formally, if *U*(*x* + *k*) is the utility and positive emotions associated with gain *k* and *U*(*x* *− k*) the utility and negative emotion associated with loss *k* with respect to the same reference point *x*, then:

1 $$\left| {U\left( {x - k} \right)} \right| \, > \, \left| {U\left( {x + k} \right)} \right|$$

Equation (1) suggests that negative emotions of unexpected losses (when one expected *x* and the results are *x-k*) are larger than the positive emotions of unexpected wins (when one expects *x* and the results are *x* + *k*). The key hypothesis we can draw from Eq. (1) is that unexpected losses should have a larger negative effect on mood and thus on fertility compared to the positive effect of unexpected wins. In other words, we can expect that the negative mood shock induced by unexpected losses is larger than the positive mood shock linked to unexpected wins.

There are two empirical challenges when it comes to testing the model of reference-dependent utility and, thus, to investigating the effect of mood on individual behavior. To start with, the reference point *x* for expectations is typically unobservable. Moreover, variation around the reference point must be orthogonal to other determinants of the behavior under study, in our case fertility. We discuss the details of our identification strategy to overcome these challenges (i.e., the unobservability of *x* and endogeneity of the factors producing gain and losses *k*), in the next section.

## Data, Variables and Model

We employ three sources of data. First, we use the Spanish Birth registers that include data on the universe of births in Spain. From these registers, we compute the monthly counts of births, by province, between 2001 and 2015.^3^ Second, we collect data from Internet on betting odds and the actual outcomes of every game of the Spanish major soccer league (*la Liga*) from season 2000/2001 to season 2014/2015.^4^ Finally, we use survey data by the Spanish Center for Sociological Research (CIS), and in particular a special survey devoted to sports conducted in 2014 (CIS 2014), to measure the intensity of support for different soccer teams in each province.

Our unit of analysis is the province. The *dependent variable* is the logarithm of the number of births in a given province and month. The key *independent variables* are then the number of predicted and unpredicted wins and losses of the most popular soccer team that is based in that province nine months before. To construct these variables, we repurpose betting odds prior to games in the Spanish soccer *Liga* and consider them as the observable reference point for individual expectations concerning the outcomes of the games (Salganik 2018). The comparison between betting odds and actual soccer games outcomes is the key variable we use to define unexpected outcomes that might trigger variations in mood, the likelihood of sexual intercourse and subsequently the number of births nine months thereafter (Card and Dahl 2011; Healy et al. 2010). In assigning the number of wins and losses to the province-by-month specific number of births, we also adjust for gestational age in order to consider births that ended up in a preterm delivery. For each province and month, we then compute the number of matches that ended in an: (1) expected win; (2) unexpected win; (3) expected loss; (4) unexpected loss.

Since in some cases^5^ (*n* = 8) there are two or more teams based in the same province, we consider the most popular one, drawing on data from CIS (2014). Based on betting odds and games outcomes, we classify the games as predicted losses (*p* < 1/3), predicted close (*p* > 1/3 and *p* < 2/3) and predicted wins (*p* > 2/3), where *p* is defined as the reciprocal of the decimal odds for a victory. Unexpected wins are predicted losses that end up in victory, and unexpected losses are predicted wins that end up in loss. We also replicate the analysis using a more extreme definition of predicted losses (*p* < 1/4) and wins (*p* > 4/5).

Formally, our baseline regression is the following

2 $${\text{Log}} \left( {{\text{Births}}} \right)_{pt} = \gamma_{p} + \delta_{t} + \beta_{1} {\text{Predicted}}\,{\text{Losses}}_{{p,t - {9}}} + \beta_{2} {\text{Unexpected}}\,{\text{Wins}}_{p,t - 9} + \beta_{3} {\text{Predicted}}\,{\text{Wins}}_{p,t - 9} + \beta_{4} \,{\text{Unexpected}}\,{\text{Losses}}_{p,t - 9} + u_{p,t}$$

where ${\text{Predicted}}\,{\text{Losses}}_{p,t - 9}$ is the number of predicted losses for the major team of the province *p* that occurred in the month *t* − 9, i.e., nine months (gestational age-adjusted) before month *t*; the remaining variables are defined likewise. The coefficients of interests for the reference utility model and prospect theory are $\beta_{2}$ and $\beta_{4}$ because they refer to unexpected results that might foster mood shocks. Conversely, predicted losses or wins should not affect mood and therefore $\beta_{1}$ and $\beta_{3}$ should be equal to 0. Note, however, that an explanation in terms of celebratory intercourse (or sorrowful abstention) could still imply a positive effect of expected wins (losses), as supporters might equally celebrate or suffer due to an expected outcome. Finally, according to prospect theory unexpected losses should matter more than unexpected wins, and therefore, one can additionally hypothesize that $\beta_{4}$ is larger in absolute value than $\beta_{2}$.

Note that we include province fixed effects ($\gamma_{{\text{p}}} )$ to deal with systematic differences in fertility and soccer results across provinces (and hence, outcomes across teams) and time (month by year, $\delta_{{\text{t}}}$) fixed effects to account for seasonality as well as for any time-specific nation-wide shocks to fertility or soccer outcomes. Note also that we include summer months when there are no soccer games and provinces that had no local team playing the Spanish major national soccer league (la *Liga*). These province by month observations have values 0 on all the four variables that refer to losses and wins but still contribute to fixed effect year estimation.

Table 1 displays descriptive statistics.^6^ We have data for 52 provinces for 13 full years (2002–2014) and one-half year (2001) because betting odds data are available from September 2000 to 2001. The total number of “provinces by month” observations is thus 8476. The number of unexpected losses and wins is not the same because an unexpected win of a team does not always correspond to an expected loss of the opponent, since betting odds also foresee draws as outcomes. The variable ‘level of support’ measures the proportion of people in a given province who declare support for the local team. The average level of support is 0.35. Note that in those provinces that do not have a team playing in the *Liga* this variable is coded as 0.

**Table 1:** Descriptive statistics. *Source*: Spanish Birth registry for log monthly births; CIS (2014) for level of support; betting odds and actual results of the Spanish Liga between 2001 and 2015 (see main text)

	*N*	Mean	SD	Min	Max
Log monthly births	8476	7.17	1.08	3.66	8.87
Monthly births	8476	2191.23	2107.73	39	7084
Expected wins	8476	0.57	1.10	0	6
Expected losses	8476	0.35	0.80	0	6
Unexpected wins	8476	0.07	0.29	0	4
Unexpected losses	8476	0.05	0.23	0	3
Monthly games	8476	1.77	2.02	0	6
Level of support	8476	0.35	0.27	0	0.75
Year	8476	2007.70	3.93	2001	2014
Month	8476	6.61	3.44	1	12

Weighted by average births by province

## Identification Assumptions

The identification assumption for a causal interpretation of our model is that betting odds incorporate all the relevant information before the game, with deviations from predicted outcomes being random shocks. The very high predictive power of betting markets suggests that this assumption is indeed plausible. This corresponds to an ignorability assumption where there are no other unobserved confounders that jointly affect deviations from predicted outcomes in betting markets and fertility. Other papers that relied on the same assumption include Card and Dahl (2011), Healy et al. (2010) and Munyo and Rossi (2013).

An additional assumption for our interpretation of the results is that unexpected soccer results trigger a mood shock on a sizeable segment of the population. We refer to this assumption as relevance assumption. Soccer is by far the most popular sport in Spain. Survey data indicate that about 70% of Spaniards age 18–44 declare to be close to, or have a sympathy for, a football team (CIS 2014). Among those who express support for a team, 70% watch its matches on TV “whenever they can” and 50% keep a flag or object related to the team at home. The large number of people who identify with a football team and follow its matches in Spain makes our relevance assumption plausible. Moreover, the proportion of individuals that express support and involvement for a team is higher among men. Spanish men are therefore more prone to mood shocks induced by soccer results than women.

## Results

Table 2 reports the baseline estimates. In Model 1, we control for province and time fixed effects and find that one unexpected loss by the local team (a defeat when the most popular team in the province was predicted to win with a probability > 0.66) leads, approximately, to a 0.8% decrease in the number of birth nine months later. However, we do not find any statistically significant effect for unexpected wins, with the point estimates being close to zero. This asymmetry in the effects of unpredicted losses and wins is in line with the prediction derived prospect theory that the negative mood shock of an unpredicted loss is larger than the positive mood shock of an unpredicted win.

**Table 2:** OLS regression of log monthly birth at provincial level at month *t-*9 (gestational age adjusted)

	(1)	(2)	(3)
Predicted wins at *t*-9	−0.00276	−0.00491^**^	−0.00160^*^
	(0.00199)	(0.00191)	(0.000944)
Unexpected losses at *t*-9	−0.00778^***^	−0.00681^**^	−0.00600^**^
	(0.00287)	(0.00312)	(0.00263)
Predicted losses at *t*-9	0.00433	0.00221	−0.00155
	(0.00495)	(0.00390)	(0.00197)
Unexpected wins at *t*-9	−0.00104	−0.000970	0.000355
	(0.00259)	(0.00250)	(0.00242)
Province FE	X	X	X
Month *x* Year FE	X	X	X
Province-specific trends		X	X
Month *x* Province FE			X
*N*	8476	8476	8476

Estimates weighted by average births by province

Standard errors, clustered by province, in parentheses

*p < 0.10; **p < 0.05; ***p < 0.01

The effect is, however, small in size and, for example, in the province of Madrid it would correspond to a decrease of 49 births nine months after one unexpected loss in a given month.

The results for the unexpected losses are rather stable across more restrictive specifications, including province-specific trends and month by province fixed effects (Models 2 and 3). One should also note that in these last specifications we also find a small negative effect for predicted wins. The effect is smaller in size compared to the effect of unexpected losses and very close to 0 in the last specification (Model 3). Still, this result suggests that other factors could be at play and mood shocks are only one of the possible mechanisms linking sports events and fertility. It could be, for instance, that certain supporters (the so-called fair-weather fans) are more keen to follow soccer games with friends and outside the home and get distracted from other activities, sexual intercourse included, when their team is doing particularly well and expected wins become more common. In other words, the negative effect of predicted wins could capture an increased interest for soccer games by supporters of well-performing teams that might also have a (very) small detrimental effect for fertility.^7^

An important concern about our results is whether they simply reflect some random noise in the data. For instance, Fowler and Montagnes (2015) questioned the findings that suggested that college football wins increase incumbent vote shares and tested a number of auxiliary hypotheses. Contrary to what one should expect if the original claim were true, they found that the purported effect of college football results on incumbent voting share is stronger in counties where people are less interested in college football. Following the same logic of implication analysis (Lieberson and Horwich 2008), we make three auxiliary tests.^8^

First, we replicate the main analysis adopting a more restrictive definition of predicted wins and losses, where predicted wins correspond to *p* > 4/5 and predicted losses to *p* < 1/5. The rationale of this test is that the more unexpected the outcome is, the larger the mood shock and its effect on fertility will be. Second, we restrict the analysis to games played at the end of season in May. The logic of this test is that more salient games are played in May, when important outcomes (success, access FIFA and UEFA championships, relegation) are often determined. We would then expect to observe larger effects for these games, in addition to uncertainties already captured by betting odds. Table 3 reports the results of these first two auxiliary tests. Both in the case of very unexpected outcomes (model 1–3) and in that of more salient games (model 4–6), we find that only unexpected losses influence the number of births, depressing births of about 1.3%. The effects are stronger than in the baseline specification of Table 2, as one would expect according to our proposed interpretation based on prospect theory.

**Table 3:** OLS regression of log monthly birth at the province level, by very unexpected outcomes and matches at the end of the season

	Very unexpected outcomes			Salient games		
	(1)	(2)	(3)	(4)	(5)	(6)
Predicted wins	−0.00375	−0.00524^***^	−0.00207	0.00229	0.00313	0.00313
	(0.00296)	(0.00193)	(0.00307)	(0.00334)	(0.00380)	(0.00380)
Unexpected losses	−0.0153^***^	−0.0137^***^	−0.0105^***^	−0.0118^**^	−0.0131^***^	−0.0131^***^
	(0.00254)	(0.00157)	(0.00233)	(0.00445)	(0.00462)	(0.00462)
Predicted losses	0.00475	0.00376	−0.00145	0.00766	0.00537	0.00537
	(0.00449)	(0.00441)	(0.00157)	(0.00435)	(0.00424)	(0.00424)
Unexpected wins	−0.00598	−0.00310	−0.00275	−0.00614	−0.00377	−0.00377
	(0.00587)	(0.00501)	(0.00363)	(0.00495)	(0.00532)	(0.00532)
Province FE	X	X	X	X	X	X
Month *x* Year FE	X	X	X	X	X	X
Province-specific trends		X	X		X	X
Month *x* province FE			X			X
*N*	8476	8476	8476	676	676	676

Standard errors, clustered by province, in parentheses. **p < 0.05; ***p < 0.01

Estimates weighted by average births by province

Expected win if the reciprocal of the winning odd is larger than 4/5, expected loss if smaller than 1/5

Salient games include only games played in May

Third, in Table 4 we study whether the effect of unexpected losses and wins on fertility is stronger in those provinces where the proportions of supporters of the local team are larger. To this end, we first run specifications in which we interact the main variables for unexpected wins and losses with the percentage of supporters in the province based on data from (CIS 2014) (Table 4). The logic of this additional analysis is that in provinces with a larger proportion of supporters, the exposure to the mood shock associated with an unexpected loss should be larger and that should translate into a larger reduction in the number of births. The results show that the effect of unexpected losses is stronger in those provinces with a large proportion of supporters and are, therefore, consistent with our proposed auxiliary hypothesis (although in our most restrictive specifications the interaction term is reduced and is not statistically different from 0).

**Table 4:** OLS regression of log monthly birth at the province level, by predicted and unpredicted win and losses of soccer games with the interaction with percentage of local supporters

	(1)	(2)	(3)
Unexpected losses	0.0208	0.0204	0.00355
	(0.0132)	(0.0145)	(0.0157)
Unexpected wins	−0.000678	0.000375	0.00133
	(0.00416)	(0.00401)	(0.00392)
Unexpected losses *x*% supporters	−0.0650^**^	−0.0619^*^	−0.0222
	(0.0294)	(0.0318)	(0.0355)
Unexpected wins *x*% supporters	−0.000690	−0.00493	−0.00446
	(0.0181)	(0.0167)	(0.0137)
Province FE	X	X	X
Month *x* year FE	X	X	X
Province-specific trends		X	X
Month *x* province FE			X
*N*	8476	8476	8476

Standard errors, clustered by province, in parentheses. *p < 0.10; **p < 0.05; ***p < 0.01

Estimates weighted by average births by province

All regressions control for expected outcomes and is interaction with % Supporters

### Robustness Checks

We have conducted a series of placebo tests, considering the effect of expected and unexpected wins and losses in soccer games in month *t* on number of births in months *t* + 1 to *t* + 7 and in months *t* + 10 to *t* + 13. Table 6 in “Appendix” shows that we do not find any effect for unexpected losses in any of the placebo tests. These results add credibility to our main findings.

## Conclusions

Previous studies have documented that seemingly irrelevant events may have important consequences for political preferences and opinions, for risk-taking economic decisions and for episodes of crime and violence (Card and Dahl 2011; Edmans et al. 2007; Healy et al. 2010; Munyo and Rossi 2013). These findings have been interpreted as evidence that changes in mood spread to otherwise unrelated dimensions such as evaluation of politics or of economic risk and can trigger other types of behaviors. In this article, we build on this literature and test the hypothesis that mood shocks might influence fertility behavior. To this end, we analyze the universe of births data in Spain between 2001 and 2015 and focus on mood shocks arising from soccer scores in Spain. We compare betting odds and actual outcomes of soccer games in Spain to identify exogenous mood shocks around expected outcomes.

Two previous academic articles on the effect of sport events on fertility have produced contradictory findings. The anecdotal claim of an “Iniesta generation” following the last-minute goal by the Barcelona midfielder in the UEFA Champions league semifinal against Chelsea is confirmed by Montesinos et al. (2013), while no evidence of “Super Bowl Babies” is found by Hayward and Rybińska (2017). What these two studies have in common is that they both focus on the supposed positive effect of success in a major sport event on fertility. In our study, we enlarge the explicative framework to also consider the consequence of losses. We find that an unexpected loss by the most popular soccer team in a Spanish province leads to a reduction of 0.8% in the number of births nine months later in that province. We do not find an opposite effect for unexpected wins. This finding is consistent with an asymmetric hypothesis drawn from prospect theory, stating that mood changes arise due to deviations from expected outcomes, with losses having larger effect than wins. A possible way to reconcile our findings and those by Hayward and Rybińska (2017) and Montesinos et al. (2013) is that a sport victory has to come as really unexpected with an unique collective celebration to produce an increase in the number of births, as it might have been the case for the agonic victory of FC Barcelona against Chelsea, associated with the Iniesta generation, and less so for the Super Bowl games whose outcomes tend to be more equalized a priori.

From a quantitative point of view, the point estimate of our main finding is very small. For instance, the 0.8 percent reduction in the number of births in a given province associated with one unexpected loss of the local soccer in team nine months earlier that we have documented corresponds on average to a reduction of about 49 births for each unexpected loss in a given month for the province of Madrid. The estimated effect, therefore, does not entail any consequences for the aggregate fertility rate in Spanish provinces. The decrease in the number of births nine months after an unexpected loss by the local team is likely to be compensated in the following months, by those couples who were planning to have a child. Even small-sized effect can, however, entail theoretical relevance (Elliott and Granger 2004; Bernardi et al. 2017). First, our key finding supports the idea that emotions and mood can be important determinants for fertility. Scholars should then consider how to include emotions into the increasingly popular models of planned behavior to study fertility (Ajzen and Klobas 2013; Mencarini et al. 2015). Work in close-by disciplines can provide some fruitful interdisciplinary inspiration in that direction (Elster, 1998; Massey, 2002). Second, our main finding also provides support for the prediction of prospect theory beyond its most common applications in finance, insurance and consumption-saving decisions (Barberis, 2013).

Methodologically, our study adds to a body of studies that have investigated the effect of subjective well-being on fertility. Moods and emotions are an important component of subjective well-being (Diener et al. 1999). There is now some evidence that happier people are more likely to have children and conversely that stress and poor mood might cause infertility (Aassve et al. 2012; Cetre et al. 2016; Greil 1997; Le Moglie et al. 2015; Parr 2010). Although our results refer only to short-lived mood shock, they provide critical evidence that supports a causal interpretation of the association previously found between happiness and fertility.

A major limitation of our study that makes us interpret these suggestive results with caution is that we cannot observe the intervening mechanisms between soccer scores and mood shock and between the latter and reduction in number of births. In a direct extension of this work, one could measure mood shocks with a sentiment analysis using Twitter data on province base (Mencarini et al. 2019). One could also focus on the intervening mechanism between mood shocks and fertility, i.e., reduction in sexual desire and intercourse. One could then study the effect of mood shock on some proxies for sexual arousal and intercourse, such as the internet access to porn sites (Markey and Markey 2010) or consumption of condoms and morning-after pills.

Still, these additional analyses with different indicators for mood shocks and proxies for sexual intercourse at the province level would still suffer from a major limitation that we also face in this current work, namely that we use macro-level data to test a micro-level mechanism. In this respect, future research could focus on physiological mechanisms (Bernhardt et al. 1998; van der Meij el al. 2012) and test whether testosterone change following vicarious experience of unexpected wins and losses is indeed asymmetric, so that a hormone change after unexpected losses is larger than the increase after unexpected wins. One could also look at variations in sexual interest and behaviors (Bancroft et al. 2003; Janssen et al. 2013) and analyze whether and how mood shocks related to soccer outcomes (or any other event that might affect mood) affect sexual interest and intercourse.

## Appendix

See Tables 5 and 6.

**Table 5:** OLS regression of log monthly birth at provincial level using binary indicators for football outcomes

	(1)	(2)	(3)
Expected wins (binary)	−0.00724	−0.00721	0.000963
	(0.00523)	(0.00573)	(0.00251)
Unexpected loss (binary)	−0.00761^**^	−0.00733^**^	−0.00643^**^
	(0.00298)	(0.00335)	(0.00294)
Expected loss (binary)	0.00786	0.00694	0.00172
	(0.00488)	(0.00450)	(0.00329)
Unexpected wins (binary)	0.000529	−0.000526	−0.000548
	(0.00359)	(0.00332)	(0.00307)
Province FE	X	X	X
Month *x* year FE	X	X	X
Province-specific trends		X	X
Month *x* province FE			X
*N*	8476	8476	8476

Standard errors, clustered by province, in parentheses. **p* < 0.10; ***p* < 0.05; ****p* < 0.01

Estimates weighted by average births by province

**Table 6:** OLS regression of log monthly birth at provincial level at time t + x on soccer games results at time t, placebo estimations

	*t* + 1	*t* + 2	*t* + 3	*t* + 4	*t* + 5	*t* + 6	*t* + 7	*t* + 10	t + 11	t + 12	t + 13
Predicted wins	0.000820	0.000455	−0.00104	−0.000531	0.00113	−0.000729	−0.000219	−0.000767	0.000153	−0.000531	−0.000167
	(0.000815)	(0.00114)	(0.00207)	(0.00100)	(0.00103)	(0.00101)	(0.00129)	(0.00113)	(0.00120)	(0.00100)	(0.00174)
Unexpected losses	−0.00130	0.000362	−0.00126	−0.00195	0.00156	−0.00226	0.00132	0.000583	−0.0000835	−0.00195	0.0000128
	(0.00324)	(0.00224)	(0.00242)	(0.00315)	(0.00278)	(0.00381)	(0.00266)	(0.00196)	(0.00117)	(0.00315)	(0.00185)
Predicted losses	−0.00362^**^	−0.00205	−0.00289^*^	−0.00155	−0.00314	−0.00182	−0.00247	−0.00108	0.00115	−0.00155	−0.00270
	(0.00160)	(0.00190)	(0.00153)	(0.00158)	(0.00204)	(0.00145)	(0.00171)	(0.00179)	(0.00166)	(0.00158)	(0.00185)
Unexpected wins	0.00176	0.00262	0.00279	0.00221	0.00444^**^	0.00151	−0.0000616	0.00249	0.00111	0.00221	0.00656^**^
	(0.00221)	(0.00219)	(0.00339)	(0.00231)	(0.00195)	(0.00301)	(0.00236)	(0.00246)	(0.00219)	(0.00231)	(0.00246)
*N*	8476	8476	8476	8476	8476	8476	8476	8476	8,476	8,476	8,476

Standard errors, clustered by province, in parentheses. **p* < 0.10; ***p* < 0.05; ****p* < 0.01

Estimates weighted by average births by province

## Footnotes

1 For instance, https://goal.blogs.nytimes.com/2010/01/28/the-iniesta-generation/.

2 For instance, https://www.youtube.com/watch?v=9KqekigARfE.

3 Data on births are registered since 1975, and they are available at the following address: https://www.ine.es/dyngs/INEbase/en/operacion.htm?c=Estadistica_C&cid=1254736177007&menu=resultados&secc=1254736195443&idp=1254735573002#!tabs-1254736195443.

4 The data on betting odds are available online at http://www.football-data.co.uk.

5 Provinces having more than one team are: Madrid, Barcelona, Sevilla, Cadiz, Gipuzkoa, Alicante, Valencia and Asturias. Since three provinces have teams equally popular that never played in the Spanish major soccer league at the same time in the period considered, we keep all teams. These provinces are Cadiz, Gipuzkoa and Asturias. We drop teams in Sevilla as they are equally popular and were rivals in the major soccer league in the period considered.

6 The replication files for the analyses presented in the article are available at the author’s personal webpage: https://www.fabriziobernardi.net.

7 This latter interpretation seems also to be supported by the results of a model where we use a binary variable for whether a team experienced any one of the four types of games in a given month. In this specification, we still find a negative effect of having experienced at least one unexpected loss in a given month, but there is no effect for predicted wins (Table 5 in “Appendix”). While the hypothesized effect of unexpected losses based on prospect theory could also be visible after one punctual mood shock, in the case of predicted wins a dummy variable is less suited to depict the “fair-weather fan” effect and distraction effect of well-performing teams (which is likely to happen after a series of positive matches).

8 We have also investigated whether some sociodemographic groups are more responsive to soccer outcomes. To do so, we have replicated our analyses separately for different subgroups, using the logarithm of the monthly number of newborns of mothers and fathers from different socioeconomic background and age-groups. We find that the effect of unexpected losses is concentrated among mid- and low-SES fathers and mothers, whereas we do not find any age-specific pattern. At the same time, using survey data we do not find any clear pattern in the likelihood of being a soccer support by SES and age-group (CIS 2014). We prefer to avoid overinterpreting these additional results (that are available upon request).
